# Supplementary material for: Coverage of completion of four ANC visits based on recommended time schedule in Northern Ethiopia: A community-based cross-sectional study design
Source: PLoS One. 2020 Aug 18;15(8):e0236965. doi: 10.1371/journal.pone.0236965 (PMC7437465; doi:10.1371/journal.pone.0236965)
Supplement: Attachment — Submitted filename: Response to Reviewrs.docx [file pone.0236965.s002.docx]

**Subject: Author’s response to reviews and editors**

**Title:** Coverage of completion of four ANC visits based on recommended time schedule in Northern Ethiopia: a community-based cross-sectional study design

**Manuscript number:** PONE-D-20-10078

**Version:** 1 **Date:** 28 Jun 2020

**Author’s response:**

Dear Editor and reviewers

Thank you for your constructive comments which help us to improve quality of our manuscript.

We have tried to address each comments raised by the editor and reviewers. Detail explanations and improvement of the revised manuscript included in the point by point response and Revised manuscript track change and manuscript without track of manuscript attached in the supplementary file.

**Dear editor:**

1. Please ensure that your manuscript meets PLOS ONE's style requirements, including those for file naming.

**Authors’ response:** thank you for your suggestion, we have tried to include the remaining style requirement of the manuscript including the file name of the documents

2. Please update both your Methods section and Ethics Statement to state whether informed participant consent was given, or a statement declaring that the institutional ethics board waived the need for consent. Please state what type of consent was given, i.e. written, verbal, etc.

**Authors’ response:** Thank you for your wonderful concern. Verbal consent was obtained from the participants. The use of verbal consent was approved by the Ethics committee.

3. We note that you have uploaded two tables both labelled table 6.  Please can you re-number your tables so that only one table is table 6. Please also ensure that you refer the renumbered Tables in your text; if accepted, production will need this reference to link the reader to the Table.

**Authors’ response:** Thank you for your crucial suggestions, based on your suggestion we tried to rearrange the tables

**Editor concern on the table 3**

**Authors’ response:** Thank you for your comment. We tried to incorporate your concern in the revised manuscript

Responses to comments from Reviewer **#**1

The authors sought to perform a report on antenatal care completion in a rural area of Ethiopia. The study is well conducted; I would suggest the following revisions.

**Author’s response**: Thank you very much for acknowledging our work
Abstract
**Comment 1:** Page 1, line 30: In the section "results", I would add this concept after the first sentence: "63.9% attended four visits or more regardless the recommended time schedule".

**Authors’ response:** Thank you for your comment, we have rewritten tried to incorporate your suggestion in the revised manuscript.
**Comment 2:** Page 1 line 37: Please correct the syntax: In summary, only one of ten women attended the four ANC contacts at the recommended gestational age. Being a member of health insurance, distance to the health facility, workload and male support were associated with the completion of four ANC visits.

**Authors’ response:** Thank you for your comment. We have rewritten your suggestion.
**Comment 3:** in the Keywords, I would substitute "recommended" with "recommended gestational age"

**Authors’ response:** Thank you for your comment, We changed and rewritten as "recommended gestational age".
**Comment 4:** Introduction
I re-wrote the introduction from line 41 to 71, mainly for syntax and word choices.
"According to WHO, Antenatal Care (ANC) can be defined as the care provided by skilled health care providers to a woman from the beginning of pregnancy until the onset of labor (1). It is a key entry point for pregnant women in order to receive a broad range of health promotion and preventive services, including screening for complications during pregnancy, delivery and postpartum period (2).
In 2003 the World Health Organization (WHO) recommended that, in low-income countries, pregnant women without any complication should visit the health facility at least four times based on recommended schedules in their pregnancy period (3). This was called Focused Antenatal care (FANC) model.
The aim was to address evidence-based clinical interventions, counseling on maternal health, on birth and emergency management (4). Even though, globally, it is reported that 83% of pregnant women received ANC at least once in their pregnancy period (5), some studies suggested that in low-income countries settings pregnant women often do not receive the recommended number of ANC visits (3). For example, the report on Sub Saharan Africa pointed that 71% of pregnant women attended formal ANC at least once; however, only 44% attended ANC four or more times (5).
Similarly, a review of trends over the last two decades has shown high variability in the proportion of pregnant women making at least four ANC visits across the globe (6).
In 2016, the WHO reviewed the previous guideline, recommending at least eight ANC visits throughout the pregnancy as a means to decrease perinatal death and enhance maternal satisfaction, irrespective of the resource setting, compared to the FANC model. However, in the real world scenario, pregnant women are not even adhering to the FANC model. Thus, the first step could be trying to reach the four ANC visits goal.
In Ethiopia, although ANC services are provided in many places (community health post, health centers and hospitals), only 27% of rural women have four or more ANC visits (8). In previous studies, level of education, ANC knowledge, service satisfaction, maternal age, parity and correct communication of pregnancy timing were found to be contributing factors for the completion of four ANC visits (9). Women who have an inadequate number of ANC visits or start ANC later than the first trimester seem to have higher rates of poor pregnancy outcomes, such as low birth weight and preterm birth. Completing four ANC visits is pivotal in fostering early (first trimester) involvement, safe delivery and postnatal care attendance among women in low-income countries (1)."

**Authors’ response:** Really very thanks for your comprehensive and crucial comments, which helps in improving the quality of the manuscript. Based on your suggestion we have rewritten the introduction part.
**Comment 5:** Study design Page 2, Line 95: I would explain what is "social desirability bias". I think it is not obvious for an audience of obstetrics and gynecologists, maybe more easy to understand for psychologists.
**Authors’ response:** Thank you for your good insight, we have corrected and rewritten as, to complement the quantitative evidence, we collected qualitative data from mothers other than who participated in the quantitative study.
**Comment 6:** Results
- Page 5, lines 175-177: please write n=423, 90.8%, n=443, 95.1% and n=366, 78.5%.

**Authors’ response:** We agree with your comment. We have rewritten
**Comment 7:** Page 5, table 1: what does it mean "Maternal occupation: employed"? What type of job? Please specify.

**Authors’ response:** Thank you for your concern. The variable Employed includes private and governmental employment

**Comment 8:** Page 9 table 5: how did you define the dichotomic variable: "mother's workload at home"? Please specify also here (it is better explained at page 11 lines 262-266).

**Authors’ response:** Thank you for your concern. The mother’s workload at home, simply collected from mothers by asking “do you have a workload at home to the extent of affecting of ANC visit based on the recommended time schedule and have two options either “Yes” or ”No”. Since this might be exposed for subjectivity, we tried to complement by the qualitative part.

Discussion

**Comment 9:** I was impressed by this concept: the prevalence of women attending the four ANC visits at recommended gestational age (GA) is 9.9%. However, 63.9% attended four visits or more regardless the recommended time schedule. At a first glance one might think that the main issue is attending visits, regardless the GA. But then, I focused on the fact that the majority of population attends four visits, but the issue is the correct timing. Maybe health care providers should stress during the first visit the importance of the correct timing of obstetrical checks. I would relate this to two important variables in Table 6: 26.4% did not receive counseling about completing four visits and 63.5% waited more than 30 minutes in the health facility. I argue that an improvement of these two components of ANC services could lead to better adhesion to 4 ANC visits. Please expand on this.

**Authors’ response:** Thank you for your good insight, been receiving counseling does not guarantee for the visit based on the recommended time schedule. This could be probably due to poor quality of ANC counseling and another reason might be due to study participants might increase health seek behavior due to some feelings of discomfort. Another issue might be due to the availability of home based ANC by the community health workers that may interfere with the scheduled ANC visits. Study from rural Tigrai demonstrated that 59.3 % of pregnant women received ANC visit from the community health workers[1].
**Comment 10:** Page 12, Line 279- 283: remove these sentences.

**Authors’ response:** We agree with your comment. We have removed
**Comment 11:** Page 12 line 284 "compared to the study performed in Jimma Town"

**Authors’ response:** We agree with your comment and corrected

**Comment 12:** Page 12, line 286: " This might be due to the difference..."

**Authors’ response:** Thank you for your comment. We incorporated and rewritten
**Comment 13:** page 13 line 301: what do you mean with the term "utilization"?

**Authors’ response:** Thank you for your comment. In this manuscript the term utilization was used to describe the prevalence of ANC visit. We corrected the word utilization to “prevalence”

**Comment 14**: Page 13 line 310: an estimated 49% of women who had delayed first ANC visits

**Authors’ response:** We agreed your comment and we have rewritten

**Comment 15:** Page 14 line 316: Our result was also lower than...

**Authors’ response:** Thank you for your comment. We have tried to incorporate your suggestion in the revised manuscript

**Comment 16:** Page 14 lines 318-319 : did not initiate, please remove "were" and choose between "by" or "at"

**Authors’ response:** We have checked line lines 318-319 agree with you, we included at and removed the word were.

**Comment 17:** Page 14 line 343: this idea
**Authors’ response:** Thank you for your comment. We agreed and tried to incorporate your suggestion
Conclusions
**Comment 18:** Page 15 line 346: what do you mean with "plus"?

**Authors’ response:** thank you for your concern, since the prevalence of completion of four ANC visits has been just 9.9%, we used	“plus” to indicate or show “ more than” 90%	of mother were not completed	the ANC based on the recommended time schedule.
**Comment 19:** Page 15 line 352: I would substitute "reducing" with " increasing" and remove lines 353 and 354 because you have already expressed this concept. Please also remove lines 360/361

**Authors’ response:** We have checked line 352 agree with you, we corrected. Besides, we have checked line 353 and 360/361, agree with you, we omitted.

**Comment 20:** Page 15 lines 363-367: please rewrite: " This is the first study that evaluates ANC visits in the context of the correct gestational age. The methods could be reproduced in other low-income countries settings or other places in Ethiopia".
**Authors’ response:** Thank you for your suggestion. We agreed with your comment and rewritten

Limitations
**Comment 21**: Please remove lines 369-370: you already explained this concept.

**Authors’ response:** We agree with your comment and we removed.

Responses to comments from Reviewer **#**

Dear authors, I would like to thank for your concern on maternal health care service utilization .Your manuscript need major revision especially on grammatical arrangement throughout the manuscript. Beside this, the discussion part is not well organized, coherent and well sounded. See all the comments Bellow:
**Comment 1:** Line -37-40-the abstract session doesn’t show recommendation

**Authors’ response:** thank you for your comment. We have included ‘the existing health system should consider improving the recommended ANC visit by integrating Community based interventions”as a recommendation

**Comment 2:** Line 48 –new sentence should be started with capital letter after punctuation .

**Authors’ response:** We agreed with your comment and we have tried correct based on your suggestion
**Comment 3:** Line 66- ‘ health provider knowledge for early pregnancy months’ is not clear ,how could be measure it?

**Authors’ response:** Thank you for your comment. Actually, the introduction part was rearranged and modified and “ health provider knowledge of early pregnancy months” parts was omitted

**Comment 4:** Line 67-71

**Authors’ response:** If we understand your concern, this paragraph was in the introduction part and adapted from literature. We tried to modify in this way **“**Women who have inadequate number ANC visits or start ANC later than the first trimester seem to have higher rates of poor pregnancy outcomes, such as low birth weight and pre-term birth. Besides,completing four ANC visits is pivotal in fostering, safe delivery, and postnatal care attendance among women in low-income countries”
**Comment 5:** Line 76-

**Authors’ response:** We agreed with your comment. The introduction part was modified more and line 76 was omitted
**Comment 6:** Line 80- “quantitative and descriptive qualitative approach methods…. “ is there such type of study design? Try to correct it…

**Authors’ response:** We accepted your comment and rewritten as “Therefore, the aim of this study was to assess the prevalence and factors related to the completion of four ANC visit using a community based cross-sectional”

**Comment 7:** Line 95 –remove the following phrases ….‘for inclusion ‘ and complete social desirability as social desirability biases

**Authors’ response:** We have checked line 95 agree with you, we omitted the word “for inclusion”. We remove the social desirability biases from this line and modified as “to complement the quantitative evidence, we collected qualitative data from mothers other than who participated in the quantitative study”. .
**Comment 8:** Line 101 what is tabia ? is it kebele or Gotte?,you need to operationalize it or describe on the method part

**Authors’ response:** Thank you for your comment, Tabia and Kebele have similar meaning. But commonly the word Kebele is used in the Town and Tabia in the rural setting. Since our study area is in the rural we used Tabia. But not similar with Gotte. Tabia is bigger than Gotte.

**Comment 9:** . Line 119 and 128-131 have certain grammatical error.

**Authors’ response:** Thank you, we agree with you and rewritten line119 as Finally, participants were selected using simple random sampling from the registration books for women who gave birth 6 months preceding the study in the selected “Tabias and Line 128-131 as “The data was collected using a pre-tested structured questionnaire. It was prepared in English and translated into the local language (Tigrigna) and then translated back to English by language experts. Twelve data collectors and four supervisors were recruited for the data collection”.

**Comment 10:** Line 164; replace the word ‘ethics ‘with that of ethical’

**Authors’ response:** Thank you, we agree with you and we have corrected as “ethical”
**Comment 11:** Line174 needs measurement ,

**Authors’ response:** Thank you, we agree with you and we have corrected as “The median age of participant women was 27 years (±12) and an interquartile range of 22 to 34 years”

**Comment 12:** Line 176-180 -most of the figures listed on the table are repeated on the text .so , make it precise .

**Authors’ response:** Thank you, we agree with you and we have tried to incorporate your comments in the manuscript

**Comment 13:** Line 181-try to correct the heading of table 1 .Study subjects were those mothers who already gave birth 6 month prior to the study .but you considered as t says delivering mother .So, see it again including other tables and figures accordingly.

**Authors’ response:** Thank you, we agree with you and we have corrected and rewritten as Table 1 “ Socio-demographic characteristics of delivered mothers in the eastern zone of rural Tigrai Region, northern Ethiopia 2019 (n= 466)”

**Comment 14**: 182-correct the word tigrai region

**Authors’ response** : Thank you for your concern, but we can use Tigray or Tigrai interchangeably
**Comment 15:** Table one- who is literate on your study? You need to put operational definition if there is new thing otherwise those who were attending primary and secondary school should have another meaning.

**Authors’ response:** We agree with you and we have rewritten as no education, by merging illiterate and literate
**Comment 16:** Line-185-189 needs grammatical arrangement

**Authors’ response:** We agree with you and we have corrected
**Comment 17:** Table 2- what is your base to categories parity? Do you think there is no difference b/n Para one and Para two mothers on utilization and timely initiation of antenatal care?

**Authors’ response:** Thank you for your comment, though usually categorized as para one, 2-4 and ≥ 5, there are also published studies that categorize parity in both (para one, 2-4 and ≥ 5) and (para 1-2, 3-4 and > 5) [2].

**Comment 18:** On the same table (table 2) age of mothers at first pregnancy was categorized as <19 and >20 what about those mothers whose age was 19 and 20?

**Authors’ response:** Thank you for your comment, we agree with you and we have corrected as less than 20 years and greater than or equal 20 years
**Comment 19:** Line 193-197-keep its coherence

**Authors’ response:** We agree with you and corrected line 193-197 as “majority of the mothers’ source of information 368 (79%) were, from Health Extension Workers”
**Comment 20:** Table 4-correct the heading as other tables .women developmental army group leader should have 2 option s(yes /no )but here is it has 3 alternatives .s ee it carefully

**Authors’ response:** We agree with you and corrected as member of women’s development group categorized as yes or no
**Comment 21:** Line 210-be consistent headings .

**Authors’ response:** Thank you, we agree with you and we have corrected as community participation related variables
**Comment 22:** It is not related with your objective

**Authors’ response:** Thank you, we agree with you and we have corrected as community participation related variables
**Comment 23:** How did you measure uptake of components of antenatal care service ? How much service is required to say ‘ yes’ in each visit?N:B utilization of all components of ANC service can not affect ANC completion but the reveres might be true.

**Authors’ response:** Thank you, for concern. We conducted a composite score by giving minimum value of zero and maximum value one for each item. The component of ANC service was dichotomized as "yes" when participants scored above mean score value. Moreover, was categorized as "no" when participants scored below mean score value [3-5],
**Comment 25:** Line - 276 being membership in health insurance is different from health insurance plan

**Authors’ response:** Thank you, we agree with you and we have corrected as community health insurance. Because in Ethiopia, especially in the rural area the types of insurance introduced by the government so far is community health insurance.

DISCUSSION
**Comment 26:** LINE 2976-324 not in line with your objective .There is a variation b/n yours and others

**Authors’ response:** we agreed with your comment, but still there is scarcity of literature that shows the completion of four ANC visit based on the recommended time schedule. But we have tried to discuss with literature similar to our objective.

**Comment 27**: Discussion should be in line with your 2 objectives, precise and arranged coherently .But,here it includes conclusion s and back ground information about maternal health

**Authors’ response:** thank you for your comment. We agreed with your comment and corrected
**Comment 28:** Line 330-331-you are using similar citations( R-15) for a study conducted in Uganda and yem,how could be ?

**Authors’ response:** Thank you, we admitted our error and corrected
**Comment 29:** Line 334-338- try to relate your finding with others.

**Authors’ response:** thank you for your comment. Though, there is a scarcity of literature, we have tried to relate our results with other findings
**Comment 30:** Line 343 replace’ …his idea… ‘With ‘…this idea…

**Authors’ response:** thank you for your comment we corrected his idea to “this idea”
**Comment 31**: Conclusion part Line 346’-no need of repetition of result here rather you can say high or low according to others result

**Authors’ response:** Thank you for your comment, we accepted and modified
**Comment 32:** 351-358 shows the significance of the study and Justification so, remove it since it is not the right place

**Authors’ response:** Thank you for your comment, we accepted and corrected
**Comment 33**: Line 360-363the recommendations ; the recommendations part deals about awareness creation, availability and accessibility of ANC service However you didn’t show the gap on the result part .So , the recommendation should be in line with your findings, which can answer your objectives.

**Authors’ response:** We agree with your comment. We have modified
**Comment 34:** Line 370 in the limitation part recall bias was one of the problems you faced during data collection. How did you manage it? Because it directly affects your outcome variable

**Authors’ response:** we know that recall bias is problematic, especially in case control studies . However, in this study it is possible to introduce recall bias. Because the participants have been asked to recall the visits that has been conducted. To minimize the recall bias; we tried to construct the tool during pre-testing by checking the collected data by interviewing the participants and reviewing the registers of participants in their respective facilities. We, also, tried to collect in-depth information in the qualitative interview. In addition, most countres used data in DHS long time that is two years preceding the survey.

**Comment 35:** Line 371-372what was the need of excluding mothers who had a previous history of pregnancy complication ? Even it could be one of the factor to attained ANC timely .Idont think so ,it could be considered as limitation of study .

**Authors’ response:** Thank you for your concern. Since, the participants were randomly selected during the data collection, the number and time of visits may be affected by the previous history of the women. This is supported by a literature showed that, essential intervention showed can be provided over four visits at the specific interval, at list for healthy women which no underlying medical problems **[6].**
**Comment 36**: Reference –line 410,REF 13 and line 416 R16 are similar references

**Authors’ response:** Thank you for your comment. We agreed the comment and corrected
**Comment 37:** Line414 REF14 and REF17 are similar references.

**Authors’ response:** Thank you for your comment. We agreed the comment and corrected

**Reference**

1.	Tesfau, Y.B., et al., *Postnatal home visits by health extension workers in rural areas of Ethiopia: a cross-sectional study design.* BMC Pregnancy and Childbirth, 2020. **20**: p. 1-9.

2.	Kebede, A.S., I.O. Ajayi, and A.O. Arowojolu, *Effect of enhanced reminders on postnatal clinic attendance in Addis Ababa, Ethiopia: a cluster randomized controlled trial.* Global health action, 2019. **12**(1): p. 1609297.

3.	Muchie, K.F., *Quality of antenatal care services and completion of four or more antenatal care visits in Ethiopia: a finding based on a demographic and health survey.* BMC pregnancy and childbirth, 2017. **17**(1): p. 1-7.

4.	Islam, M.M. and M.S. Masud, *Determinants of frequency and contents of antenatal care visits in Bangladesh: Assessing the extent of compliance with the WHO recommendations.* PloS one, 2018. **13**(9): p. e0204752.

5.	Gupta, S., et al., *Factors associated with four or more antenatal care visits and its decline among pregnant women in Tanzania between 1999 and 2010.* PloS one, 2014. **9**(7).

6.	Lincetto, O., et al., *Antenatal care.* Opportunities for Africa's newborns: Practical data, policy and programmatic support for newborn care in Africa, 2006: p. 55-62.
